# Small Intestine Bacterial Overgrowth in Bangladeshi Infants Is Associated With Growth Stunting in a Longitudinal Cohort

**DOI:** 10.14309/ajg.0000000000001535

**Published:** 2021-10-25

**Authors:** Jeffrey R. Donowitz, Zhen Pu, Ye Lin, Masud Alam, Tahsin Ferdous, Talat Shama, Mami Taniuchi, Md Ohedul Islam, Mamun Kabir, Uma Nayak, Abu S.G. Faruque, Rashidul Haque, Jennie Z. Ma, William A. Petri

**Affiliations:** 1 Division of Pediatric Infectious Diseases, Children's Hospital of Richmond at Virginia Commonwealth University, Richmond, Virginia, USA;; 2 Division of Infectious Diseases and International Health, University of Virginia, Charlottesville, Virginia, USA;; 3 Department of Statistics, University of Virginia, Charlottesville, Virginia, USA;; 4 Infectious Diseases Division, the International Centre for Diarrhoeal Disease Research, Bangladesh, Dhaka, Bangladesh;; 5 Department of Biomedical Engineering, University of Virginia, Charlottesville, Virginia, USA;; 6 Department of Engineering Systems and Environment, University of Virginia, Charlottesville, Virginia, USA;; 7 Department of Public Health Sciences University of Virginia, Charlottesville, Virginia, USA;; 8 Department of Public Health Sciences, University of Virginia, Charlottesville, Virginia, USA;; 9 Nutrition and Clinical Services Division, the International Centre for Diarrhoeal Disease Research, Bangladesh, Dhaka, Bangladesh.

## Abstract

Small intestine bacterial overgrowth (SIBO) is common in children from low-income countries and has been cross-sectionally associated with growth stunting. We sought to determine whether SIBO was associated with poor growth and neurodevelopmental in a longitudinal analysis.

**METHODS:**

We measured SIBO by glucose hydrogen breath test (GHBT) at 18, 52, 78, and 104 weeks of life in a prospective longitudinal birth cohort of Bangladeshi children. Sociodemographic information and measures of enteric inflammation were analyzed as covariates. Diarrheal samples were tested for enteropathogens using polymerase chain reaction. Regression models were created using standardized mean GHBT area under the H_2_ curve (AUC) to determine associations with linear growth and cognitive, language, and motor scores on the Bayley-III Scales of Infant and Toddler Development at 2 years. We also investigated associations between GHBT AUC and enteropathogen exposure.

**RESULTS:**

A 1-ppm increase in standardized mean GHBT AUC was associated with a 0.01-SD decrease in length-for-age Z score (*P* = 0.03) and a 0.11-point decrease in Bayley language score (*P* = 0.05) at 2 years of age in adjusted analysis. Enteroaggregative *Escherichia coli*, Enteropathogenic *Escherichia coli, Giardia*, and *Enterocytozoon bieneusi* were associated with increased GHBT AUC, whereas *Clostridium difficile*, norovirus GI, sapovirus, rotavirus, and *Cryptosporidium* were associated with decreased GHBT AUC. None were consistent across all 4 time points.

**DISCUSSION:**

SIBO in the first 2 years of life is associated with growth stunting and decreased language ability in Bangladeshi infants and may represent a modifiable risk factor in poor growth and neurodevelopment in low-income countries.

## INTRODUCTION

Environmental enteric dysfunction (EED), a subclinical disorder of those living with poor sanitation, has been associated with growth stunting and neurodevelopmental delays (1–3). Stunting, which affects 1 in 4 children younger than 5 years, is a marker of impending morbidity and mortality (4,5). Stunting is a risk factor for increased infections including respiratory infection, diarrheal infection, and malaria (6–8). Stunting is also associated with poor neurodevelopment and increased risk of death before age 5 years (6,9–11). Enteric inflammation is the hallmark of EED, but recent work has described an intestinal dysbiosis associated with the condition (9,12,13). Vonaesch et al. (12) described a cohort of malnourished African children who underwent duodenoscopy and found 91% had small intestine bacterial overgrowth (SIBO) and that the bacteria cultured included *Streptococcus*, *Staphylococcus*, *Haemophilus*, *Neisseria*, *Moraxella*, and *Rothia* species. The stool of these children showed a similar trend with stunted children having a significant differential expression of *Lactobacillus* in the stunted group. Similarly, in a cohort of Bangladeshi children who underwent duodenoscopy, total bacterial load in the duodenum, as assessed by 16s rDNA analysis, negatively correlated with length-for-age Z (LAZ) score. *Streptococcus*, *Rothia*, and *Veillonella* species were independently associated with poor linear growth (13). The same group was able to show that a novel diet, microbiota-directed food intervention, was able to increase beneficial taxa and improve weight gain compared with standard ready-to-use supplementary food in malnourished Bangladeshi children (14).

SIBO is a particular dysbiosis which was first described in 1939 in patients with small intestine structural abnormalities and macrocytic anemia (15). Since then, SIBO has become recognized as a secondary condition which develops in the setting of impaired intestinal motility, hypochlorhydria, or anatomic intestinal defects (16). Although the gold standard for SIBO diagnosis is culture of duodenal aspirate, glucose hydrogen breath testing is a noninvasive diagnostic alternative (17). Asymptomatic school-aged children of lower socioeconomic status living in low- and middle-income countries (LMICs) have a prevalence of SIBO as high as 30% (18−21). In this setting, SIBO has been associated with poor carbohydrate absorption and underperformance of an oral cholera vaccine (22,23). Furthermore, our group showed a positive glucose hydrogen breath test (GHBT) was associated with enteric inflammation and linear growth shortfalls in a cross-sectional analysis of Bangladeshi 2-year-olds and that a positive breath test was associated with increased fecal *Lactobacillus* (18,24).

As all previous work on SIBO in children from LMICs was cross-sectional, a predictive timeline suggesting causality has never been established. Here, we report the findings from the first longitudinal study of SIBO in LMICs. We conducted a 2-year observational birth cohort study to determine whether SIBO in early childhood was predictive of linear growth delays. Our primary hypothesis was that SIBO would be a significant predictor of subsequent linear growth shortfalls. Given that EED has also been associated with neurodevelopmental delay, we hypothesized that there would also be an association between a positive GHBT and lower scores on neurodevelopmental testing.

## METHODS

### Study site and population

We conducted a longitudinal birth cohort study to investigate the effects of SIBO on linear growth (ClinicalTrials.gov identifier: NCT02745327). Children were enrolled within 7 days of birth and followed for 2 years. Enrollment ran from November, 2014, until March, 2016. This study was conducted in the densely populated urban slum areas of the Mirpur neighborhood in Dhaka, Bangladesh. The population is relatively ethnically homogenous with the majority being Bihari. More than 95% of homes are mud brick or tin. Uncovered sewer drains directly abut 59% of dwellings and 85% of families use toilet facilities shared with other households. Because of the location of our study clinic, our subjects tended to come from the lower socioeconomic stratum of Mirpur.

### Data collection

Pregnant mothers were enrolled during their third trimester. We conducted a survey of potential mothers in our catchment area, and recruitment was largely by word of mouth conducted by field research assistants who are well known in the neighborhood. At enrollment, a questionnaire was administered to mothers to collect data on socioeconomic and sanitation variables. Children were enrolled within 7 days of birth, and all healthy children without known pre-existing conditions were eligible for enrollment. Children with an enrolled sibling were excluded. Children born by both vaginal and cesarean delivery were enrolled because EED is believed to develop because of postnatally acquired enteric infection. Field research assistants made home visits twice weekly and assessed for diarrhea. A diarrheal episode was defined as >3 unformed stools in a 24-hour window and separated from a previous diarrheal episode by at least 3 days. A stool sample was taken for each diarrheal episode. Field research assistants collected a fresh stool in sterile DNase/RNase-free containers and placed the sample into coolers with ice packs in the field for transport to our study clinic where they were placed into 4^o^C. Blood was collected by a trained pediatric phlebotomist at 18, 52, 78, and 104 weeks of age. Samples were then transported each day through cooler to the Parasitology Laboratory at the International Centre for Diarrhoeal Disease Research, Bangladesh, where they were aliquoted and placed in −80 ^o^C for storage until used for biomarker analysis or total nucleic acid (TNA) extraction, both of which were batched.

SIBO was assessed through GHBT at 18, 52, 78, and 104 weeks of age. As an exploratory endeavor, children who were positive (any value > 12 ppm exhaled hydrogen over the patient's baseline value during the testing period) were given the option to return for repeat testing every 2 months until the child was negative. Because of the cumbersome nature of breath testing, this was made optional for families because we did not want to overburden participants. Children younger than 12 months were fasted for 2 hours before testing and those older than 12 months for 3 hours. Any child who was acutely ill was rescheduled for after the illness resolved. Any child who had taken antibiotics in the 14 days before a scheduled breath test was rescheduled to ensure a 14-day antibiotic-free period before testing. A baseline breath sample was collected using the Quintron (Milwaukee, WI) child breath collection bag and 1-way flutter valve which was connected to an appropriately sized pediatric anesthesia mask. Children were then given a glucose solution of 100-g glucose in 500-mL sterile water administered at 5-mL/kg body weight over 10 minutes. Breath was collected every 20 minutes for 2 hours in children younger than 12 months and for 3 hours in children older than 12 months. Samples were immediately tested for hydrogen content using a Quintron BreathTracker SC (Milwaukee, WI) gas chromatograph. Samples with lower than expected CO_2_, per the manufacturer's protocol, were considered contaminated with room air, discarded, and immediately recollected. Children were allowed only water during the testing period. Covariates measuring enteric inflammation characteristic of EED including fecal myeloperoxidase (ALPCO, Salem, NH) and Reg 1B (TechLab, Blacksburg, VA) were measured by commercially available enzyme-linked immunosorbent assay. Myeloperoxidase is a peroxidase enzyme expressed by neutrophils and, in the stool, is a marker of neutrophil infiltration in the gut. Reg 1B is an antiapoptotic, proproliferative protein secreted by damaged epithelial cells. Interleukin 4 (IL-4), a member of the Th2 signaling pathway, was also measured by enzyme-linked immunosorbent assay because of its association with improved cognitive scores in the LMIC pediatric population (ALPCO) (25).

Anthropometry was measured at enrollment for both mothers and infants. Infants were then measured every 3 months. For infants, measuring boards and calibrated scales were used by staff trained in the procedure. Maternal anthropometry was measured using calibrated scales and stadiometers (a ruler and a sliding horizontal headpiece). Neurodevelopmental assessment was made using the Bayley-III Scales of Infant and Toddler Development at the 2-year-old study visit by a psychologist trained to use the tool. The version used in this study was culturally adapted for Bangladesh but not normalized to the entire Bangladeshi population. Our group has used this tool in previous studies, and it has been determined to have high short-term (within 7 days) retest reliability (r > 0.80) and high interobserver reliability (r = 0.99) (3,25). This tool assesses cognitive, motor (fine and gross), and language (receptive and expressive) skills. Composite scores for each section were used as outcomes in our analyses.

Diarrheal stool samples were assessed for presence of fecal pathogens using a TaqMan Array Card platform which tested for 36 enteric pathogens, the methods of which have been previously published (26). Briefly, TNA was extracted from 200 mg of stool using a slightly modified protocol of the QIAamp DNA Fast Stool Mini Kit (Qiagen, Gaithersburg, MD) (27,28). TNA was stored at −80 ^o^C until testing. Quantification cycle cutoff thresholds of less than 35 were considered positive (29). A positive result was only considered valid if the corresponding extraction blank for that target was negative. A negative result was only considered valid if the positive controls were positive for the given sample. Each sample was spiked with phocine herpes virus (Erasmus MC, Department of Virology, Rotterdam, the Netherlands) and bacteriophage MS2 (ATCC 15597B; American Type Culture Collection, Manassas, VA) as extrinsic controls for extraction and amplification. *Escherichia coli* pathotypes were defined based on known virulence genes. Enteroaggregative *E. coli* (EAEC) was defined as aaiC and/or aatA, typical enteropathogenic *E. coli* (typical EPEC) as bfpA with or without eae, heat stable toxin-producing enterotoxigenic *E. coli* as STp and/or STh with or without LT, heat labile toxin-producing enterotoxigenic *E. coli* as LT only, Shiga toxin-producing *E. coli* as stx1 and/or stx2 with or without eae, and *Shigella* as ipaH. Of note, ipaH can also define enteroinvasive *E. coli* (EIEC), but in previous work delineating *Shigella* from EIEC in Bangladesh, almost no EIEC was found, so in this analysis, we assumed ipaH-positive samples to be *Shigella*.

### Statistical methods

We aimed to enroll 270 children which, based on our previous work and assuming at 10% drop out rate per year, would give us an 80% power to detect a small to medium effect size when comparing SIBO-positive and -negative children for our primary outcome of LAZ at 2 years (18).

GHBT output was initially assessed and summarized in 3 ways. The number of positive SIBO tests was the summation of positive tests where any value > 12 ppm over the patient's baseline during the testing period was considered positive and thus ranged from 0 to 4. Based on The North American Consensus Group on Hydrogen and Methane-Based Breath Testing's statement in 2017, published after our data were collected, we reanalyzed this variable using a >20-ppm cutoff which had no significant effect on our results (30). Thus, we used the >12-ppm cutoff specified in our original analysis plan for the remainder of our analyses. The maximum change in hydrogen for each test taken was averaged then standardized to the number of tests taken which resulted in standardized mean maximum H_2_ delta. Finally, the area under the hydrogen curve (AUC) was calculated as the sum of the trapezoids under the hydrogen curve. This was then averaged across tests and standardized to the number of tests taken to give the standardized mean GHBT AUC. Using only children with 3 or more GHBTs and complete covariate data sets, each of these 3 summary variables were regressed on LAZ at 2 years of age. We chose the variable with the highest R^2^, standardized mean GHBT AUC, for use our final models.

We created a multivariable linear regression model for LAZ at 2 years including income, use of treated water, presence of a septic tank/toilet, dichotomized IL-4 at week 18 (IL-4 > 0), fecal myeloperoxidase at week 18, fecal Reg 1B at week 18, sex, mother's height, maternal education (none vs any formal education), LAZ at enrollment, and standardized mean GHBT AUC. The final model was determined with stepwise selection in the regression analysis. Stepwise regression was also performed with the composite motor, language, and cognitive scores on the Bayley-III Scales of Infant and Toddler Development as the outcomes, and the same set of predictors was included in the final model for the 3 neurodevelopmental outcomes. Standardized mean GHBT AUC was retained in all models.

To further assess the effect of GHBT AUC on growth, we separated the data in the bottom and top 25^th^ percentiles for standardized mean GHBT AUC and conducted a survival analysis for the time to being stunted (i.e., time to the first LAZ measurement < −2 SD) between these 2 phenotypic extremes.

Finally, we analyzed the TaqMan Array Card data and determined whether children had a given pathogen detected in their diarrheal stool within the 3 months before each GHBT. A Mann-Whitney *U* test was performed to determine whether there was a significant difference in mean GHBT AUC between pathogen exposed and unexposed children. Each pathogen at each time point was assessed independently. Only pathogens with 5% prevalence at a given time point were included in the analysis of that time point. As this was an exploratory analysis, we did not correct for multiple comparisons. However, FDR-adjusted analyses were performed, and the results are available in Supplementary Table 1, Supplementary Digital Content 1, http://links.lww.com/AJG/C258.

### Bioethics statement

This study was approved by the Research and Ethics review committees of the International Centre for Diarrhoeal Disease Research, Bangladesh. Parents of all children signed informed consent documents that were reviewed with them by our study staff at a level appropriate to their education.

## RESULTS

**Figure 1.**
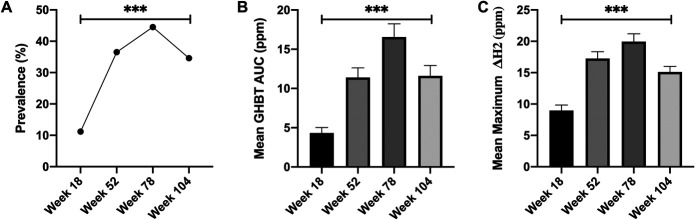
Glucose hydrogen breath test output over the first 2 years of life. We analyzed 3 separate outputs of the glucose hydrogen breath test. Prevalence (as determined by > 12 ppm over the subject's baseline) (**a**), mean (with SE shown by whiskers) maximum change in breath hydrogen from baseline (**b**), and mean (with SE shown by whiskers) area under the hydrogen curve (AUC) (**c**). All measures demonstrated an increasing trend from 18 weeks of age to 78 weeks with a slight drop at 104 weeks. Differences across time were significant for all measures (χ^2^ *P* value for prevalence and Welch's ANOVA *P* value for mean maximum change in breath hydrogen and mean GHBT AUC; all were *P* < 0.001***).

Two hundred seventeen children were enrolled. Using the dichotomous cutoff of >12 ppm over the patient's baseline for SIBO positivity, 11.2% of children (29 of 259 tested) were considered to be SIBO-positive at 18 weeks. This increased to 36.5% (85 of 233 tested) by 52 weeks, peaked at 44.5.% (97 of 218 tested) at 78 weeks, and was 34.6% (72 of 208 tested) at 104 weeks. Mean maximum H_2_ delta showed a similar trend of 4.33 ppm at 18 weeks, 11.41 ppm at 52 weeks, 16.55 ppm at 78 weeks, and 11.60 ppm at 104 weeks. Likewise, mean GHBT AUC was 8.97 ppm at 18 weeks, 17.25 ppm at 52 weeks, 19.94 ppm at 78 weeks, and 15.11 ppm at 104 weeks. For each measure, these differences were significant across the 4 measured time points (Figure 1). Using the cutoff of >12 ppm over baseline to determine positivity, children who opted to be retested at 18 weeks had a 9.5% (2 of 21) repeat positivity rate at 2 months after test. At 52 weeks, the 2-month repeat positivity rate of children who opted to be retested was 43.8% (28 of 64) with 4 children of the 12 (33.3%) retested at 4 months after test remaining positive. At 78 weeks, 36.9% (24 of 65) of children who were retested at 2 months after test were positive with 3 of the 4 (75.0%) children retested at 4 months remaining positive. At 104 weeks, 32.1% (17 of 53) of children who opted to be rested were positive at 2 months after test with 6 of 13 (46.2%) retested remaining positive at 4 months, 3 of 6 (50.0%) retested at 6 months remaining positive, and 2 of 2 (100.0%) retested at 8 months remaining positive (Supplementary Figure 1, Supplementary Digital Content 2, http://links.lww.com/AJG/C257).

**Figure 2.**
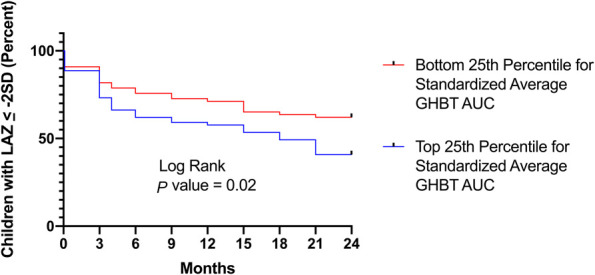
Effects of glucose hydrogen breath testing area under the H_2_ curve (AUC) on length-for-age Z score over time. Children were stratified by their standardized mean glucose hydrogen breath test (GHBT) AUC and the top and bottom quartiles compared with their time to stunting (length-for-age Z score < −2 SD). Children with higher standardized mean GHBT AUC had significantly quicker time to stunting over the first 2 years of life (*P* = 0.02).

Of the 270 enrolled, 214 had at least 3 GHBTs. The enrollment characteristics of these children did not differ from the 56 without 3 GHBTs except for 0.6 less people living per room in their homes (Table 1). Four additional children were excluded because of missing data for LAZ at 2 years leaving 210 children included in our analysis of LAZ at 2 years. In univariate analysis, 1-ppm increase in standardized mean GHBT AUC was associated with a −0.02-SD decrease in LAZ at 2 years (*P* = 0.04) with the mean standardized mean GHBT AUC in the cohort being 15.41 ppm H_2_. Total positive GHBTs over 2 years of life and standard mean maximum H_2_ delta were not significant predictors (Table 2). Our multivariable analysis with stepwise selection showed a 1-ppm increase in standardized mean GHBT AUC to be associated with a loss of 0.01 SD in LAZ at 2 years (*P* = 0.03). A 1-cm increase in mother's height was associated with an 0.04-SD increase (*P* ≤ 0.001), a 1-SD increase in LAZ at enrollment was associated with an 0.31-SD increase (*P* ≤ 0.001), and an increase of 1,000 Taka in family income was associated with an 0.16-SD increase in LAZ at 2 years (*P* = 0.001). The R^2^ of the model was 25.3% (Table 3). The likelihood to stunting from the survival analysis was significantly different in the children who were in the top quartile for standard mean GHBT AUC as compared to those in the bottom quartile (log rank *P* = 0.02) (Figure 2).

**Table 1.**
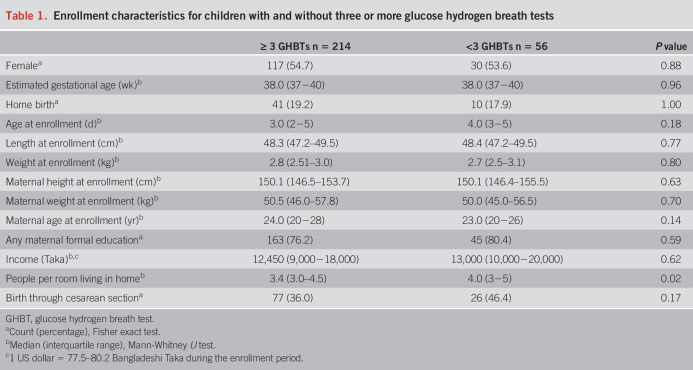
Enrollment characteristics for children with and without three or more glucose hydrogen breath tests

**Table 2.**
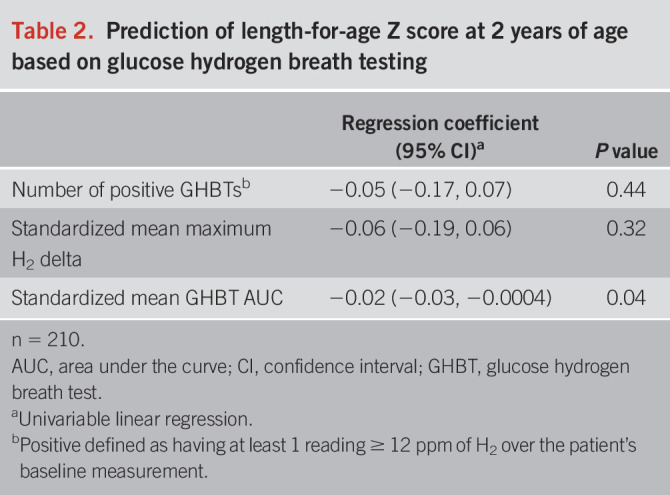
Prediction of length-for-age Z score at 2 years of age based on glucose hydrogen breath testing

**Table 3.**
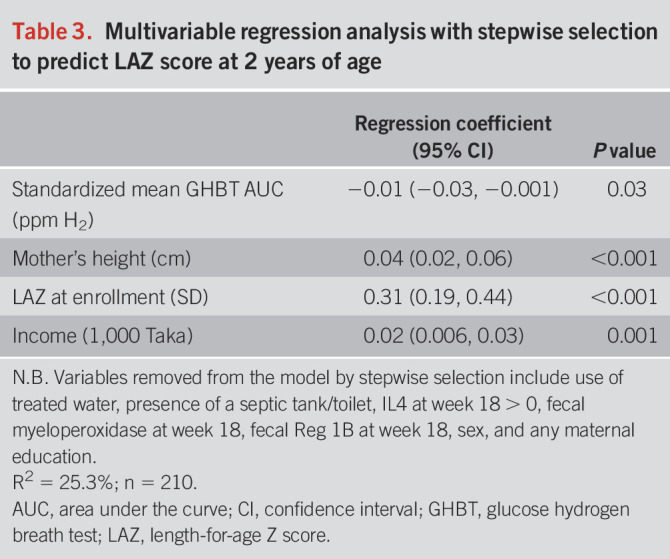
Multivariable regression analysis with stepwise selection to predict LAZ score at 2 years of age

Ten of the children with ≥ 3 GHBTs did not have Bayley testing leaving 204 for our neurodevelopmental analyses. The mean (±SD) for cognitive score was 91.0 (±7.5), for language score was 92.5 (±7.5), and for motor score was 95.0 (±9.0). In our model to predict cognitive scores, being female was associated with a 2.33-point increase (*P* = 0.02) and mothers having any formal education was associated with a 3.19-point increase (*P* = 0.006). For language score, a 1-ppm increase in standardized mean GHBT AUC was associated with a 0.11 decrease in score (*P* = 0.05). LAZ at enrollment (1.50-point increase, *P* = 0.003), use of treated water (2.10-point increase, *P* = 0.05), being female (4.74-point increase, *P* < 0.001), any maternal education (2.84-point increase, *P* = 0.009), and income (0.11-point increase per 1,000 Taka, *P* = 0.009) were associated with improved language scores. Being female (2.81-point increase, *P* = 0.02) and income (0.14-point increase per 1,000 Taka) were the only variable associated with motor score (Table 4).

**Table 4.**
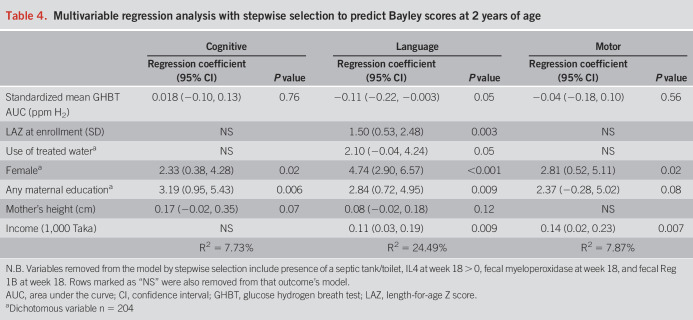
Multivariable regression analysis with stepwise selection to predict Bayley scores at 2 years of age

**Figure 3.**
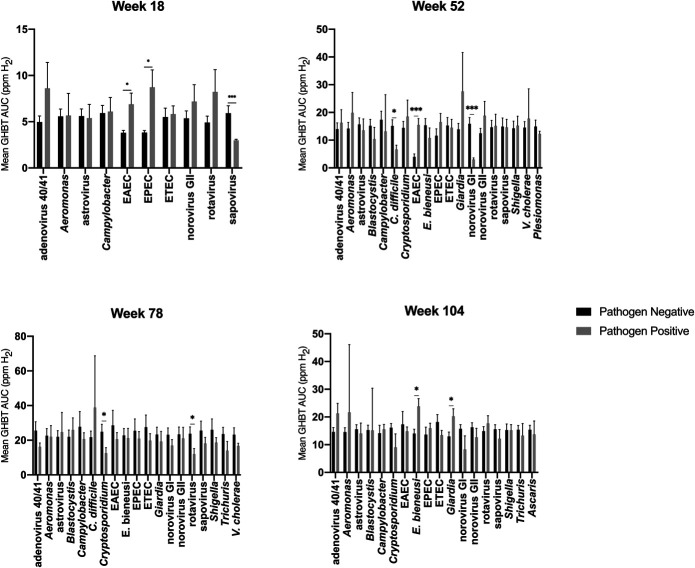
Association of pathogen carriage with glucose hydrogen breath test area under the H_2_ curve (AUC). Glucose hydrogen breath test (GHBT) AUC was compared between children with pathogens of interest in their diarrheal stool within the 3 months before each GHBT and those without. Mann-Whitney *U* tests were performed to determine whether there was a significant difference in standardized GHBT AUC between pathogen exposed and unexposed children. Each pathogen at each time point was assessed independently. * *P* value of 0.01 to 0.05, ** *P* value of 0.001 to 0.01, and *** *P* value < 0.001.

Only children with a diarrheal episode in the 3 months before a scheduled GHBT were included in the analysis of pathogen association with GHBT AUC. The GHBT AUC of these children did not differ from the excluded children except at week 18 where the excluded children had a higher GHBT AUC (9.98 vs 5.95 ppm, *P* = 0.05) (Supplementary Table 2, Supplementary Digital Content 3, http://links.lww.com/AJG/C259). At week 18, the mean GHBT AUC was increased in children who tested positive for EAEC vs children who tested negative (6.89 vs 3.81 ppm, *P* = 0.02). A similar trend was observed for EPEC (8.73 vs 3.83 ppm, *P* = 0.02). In children who tested positive for *Sapovirus*, the mean GHBT AUC was lower than in those who tested negative (2.99 vs 5.91 ppm, *P* = 0.001). At week 52, children who tested EAEC positive again had a higher mean GHBT AUC (15.53 vs 2.97 ppm, *P* < 0.001). Children who tested positive for *Clostridioides difficile* and norovirus GI had lower mean GHBT AUCs than those testing negative (6.71 vs15.15 ppm, *P* = 0.02; and 3.08 vs 15.88 ppm, *P* < 0.001, respectively). At week 78, *Cryptosporidium* (12.59 vs 24.92 ppm, *P* = 0.03) and rotavirus (12.03 vs 23.79 ppm, *P* = 0.04) exposure was associated with lower mean GHBT AUCs. At week 104, the mean GHBT AUC was higher in children who tested positive for both *Enterocytozoon bieneusi* (23.86 vs 14.02 ppm, *P* = 0.05) and *Giardia* (20.25 vs 12.97 ppm, *P* = 0.04) (Figure 3). Incidence of pathogens at each time point is available in Supplementary Table 3, Supplementary Digital Content 4, http://links.lww.com/AJG/C260.

## DISCUSSION

This work is the first to investigate SIBO through GHBT in a longitudinal study in children from a low-income setting. This expanded on cross-sectional studies reporting an association between SIBO as measured by duodenal aspirate analysis or GHBT and stunting, showing that GHBT positivity remained a risk factor for growth stunting in longitudinal analysis (12,13,18,31). In addition, we have shown a negative association between GHBT and language development. This suggests that duodenal dysbiosis may be a modifiable risk factor for growth stunting and language delay, a promising concept especially if probiotic therapies can be developed which would avoid use of antibiotics. Despite the theory that linear growth stunting and neurodevelopmental delays associated with EED stem from a common inflammatory pathway, we did not see an association of SIBO with Bayley-III scores for cognitive or motor development. Almost all children in the population studied have some evidence of EED, a process which is likely multifactorial in its pathogenesis and heterogeneous in its severity (32). This means that the background noise is high, making more subtle contributions of any 1 factor difficult to detect. Although GHBT positivity has been shown to be associated with enteric inflammation in the low-income pediatric setting, SIBO also has nutritional consequences (18). SIBO has been associated with steatorrhea and loss of fat soluble vitamins (except vitamin K which intestinal commensals produce), carbohydrate malabsorption, and a protein loosing enteropathy (16,33). The nutritional deficiency associated with SIBO may contribute more directly to growth and language delay than to cognitive and motor neurodevelopment explaining why there was no association seen with these outcomes.

Previous work has shown that SIBO in LMICs is associated with poor sanitation and markers of fecal-oral contamination of the environment (18). This suggested enteric pathogens as a possible etiology of SIBO in this setting. No pathogen demonstrated a robust association across all 4 time points. Given that this analysis was exploratory in nature, only univariate models were created. However, children in the studied population carry a mean of 3.3 enteric pathogens in nondiarrheal stool samples which means pathogens may have acted as confounders to each other in our analysis (34). It may also be that the 3-month window we investigated for presence or absence of pathogens was insufficient, especially if bacterial overgrowth is due to an autoimmune interaction with the enteric nervous system governing motility in response to a particular infection as suggested by murine models (35,36). However, the associations noted deserve further investigation and validation.

Our models used the GHBT AUC as a predictor of adverse outcomes. Although the GHBT is commonly used to diagnose SIBO, the reported sensitivity and specificity of the test when compared with endoscopically obtained culture varies widely in the reported literature leading The North American Consensus Group on Hydrogen and Methane-Based Breath Testing to state that current small bowel culture techniques are not satisfactory (30,37). The GHBT clearly identifies an upper intestinal dysbiosis involving an aberrant hydrogen economy, but the details remain unstudied and may be unique in our pediatric LMIC setting. Although not validated, the GHBT AUC has been used for research purposes and provides a continuous measure of this dysbiosis accounting for baseline hydrogen as well as response to glucose stimulation (38).

This work had several strengths. First, glucose hydrogen breath testing was longitudinal which suggests causation in the stunting pathway because SIBO was documented very early in life. Furthermore, our analysis included robust covariate measures and is the first to include neurodevelopmental assessment as a potential outcome of SIBO in the LMIC population. There are also several important limitations which should be considered when interpreting our results. First, although we had 4 GHBTs, our limited data on repeat testing demonstrated that SIBO is not a static condition and likely waxes and wanes over time. This means that 4 tests may have been insufficiently granular to capture the total time children had this dysbiosis. Although this would not have invalidated the associations we did note, it would have decreased our ability to demonstrate possible true associations that we may have missed including those with cognitive and motor neurodevelopmental scores. Second, our analysis of SIBO's association with pathogen exposure was univariate and limited to 3 months of exposure before glucose hydrogen breath testing, which may have been insufficient. Also, we only tested diarrheal stools and thus excluded children who did not have diarrhea in the 3 months before a GHBT. These excluded children were likely asymptomatic carriers of enteric pathogens which may have altered our findings. Finally, given the multifactorial nature of both infant growth and neurodevelopment, as well as the heterogeneous nature of EED, there are likely confounders we did not consider. One likely confounder we did not account for was antimicrobial use. In the Mirpur neighborhood, antibiotic use is common with a diverse range of antibiotic classes used in young children (39). These antibiotics likely influenced both the duodenal microbiota and enteric pathogen carriage during the course of our study despite our 14-day antibiotic-free window before glucose hydrogen breath testing.

Our main finding of SIBO's association with poor growth in a longitudinal analysis suggests causation, although it does not prove it. As SIBO is treatable, it is likely that it is an easily modifiable risk factor for stunting in children who continue to live in unsanitary conditions (40). Several studies have focused on identifying the particular taxa associated with SIBO and stunting in children from LMICs, although none of these has described the particular taxa associated with a positive GHBT (12,13). Further understanding of the exact nature of this small intestinal dysbiosis and the species or strains responsible for a positive breath test may lead to both the development of improved diagnostics as well as to nonantibiotic therapeutics.

## CONFLICTS OF INTEREST

**Guarantor of the article:** Jeffrey R. Donowitz, MD.

**Specific author contributions:** Study concept and design: J.D., A.S.G.F., R.H., and W.A.P.; obtained funding: J.D., A.S.G.F., and W.A.P.; acquisition of data: M.A., T.F., and T.S.; analysis and interpretation of data: J.D., Z.P., Y.L., M.T., M.O.I., M.K., and J.Z.M.; drafting of the manuscripts: J.D. and W.A.P.; critical revision of the manuscript: J.D., Z.P., YL, M.A., T.F., T.S., M.T., M.O.I., M.K., U.N., A.S.G.F., R.H., J.Z.M., and W.A.P.; study supervision: J.D., M.A., U.N., A.S.G.F., R.H., and W.A.P.

**Financial support:** This work was supported by the Pediatric Scientist Development Program [grant number 5K12HD000850] to J.D.; the National Institutes of Health [grant number 5R01AI043596 to W.A.P. and grant number 1K23HD097282 to J.D.]; and the Bill and Melinda Gates Foundation [grant number OPP1100514 to A.S.G.F.].

**Potential competing interests:** None to report.

**Study Highlights**

**WHAT IS KNOWN**

✓ Small intestine bacterial overgrowth (SIBO) is common in children from low-income countries.
✓ SIBO has been associated with growth stunting in cross-sectional analyses.

**WHAT IS NEW HERE**

✓ SIBO predicts linear growth stunting and poor language development in a longitudinal study of Bangladeshi infants.
✓ SIBO is not associated with cognitive or motor delays.
✓ SIBO is associated with enteric pathogen exposure.

## Supplementary Material

SUPPLEMENTARY MATERIAL
