# Supplementary material for: Small Intestine Bacterial Overgrowth in Bangladeshi Infants Is Associated With Growth Stunting in a Longitudinal Cohort
Source: Am J Gastroenterol. 2021 Oct 25;117(1):167–75. doi: 10.14309/ajg.0000000000001535 (PMC8715995; doi:10.14309/ajg.0000000000001535)
Supplement: SUPPLEMENTARY MATERIAL [file acg-117-167-s001.pdf]

Supplementary Figure 1. Repeat Glucose Hydrogen Breath Testing Over Time. Children who were positive at each of the 4 scheduled glucose hydrogen breath tests (GHBTs) were offered repeat testing every 2 months until negative. SIBO positivity ranged from less than 2 months to greater than 8 months. Change in enrollment numbers over time was due to subject attrition over the course of the study. Reasons for having "no result" include failure of subject to report for testing at that time point, parental refusal of GHBT at that time point, or that the test was started but stopped prematurely by the parent or study team.
