# Supplementary figures and images for: Small Intestine Bacterial Overgrowth in Bangladeshi Infants Is Associated With Growth Stunting in a Longitudinal Cohort
Source: Am J Gastroenterol. 2021 Oct 25;117(1):167–75. doi: 10.14309/ajg.0000000000001535 (PMC8715995; doi:10.14309/ajg.0000000000001535)

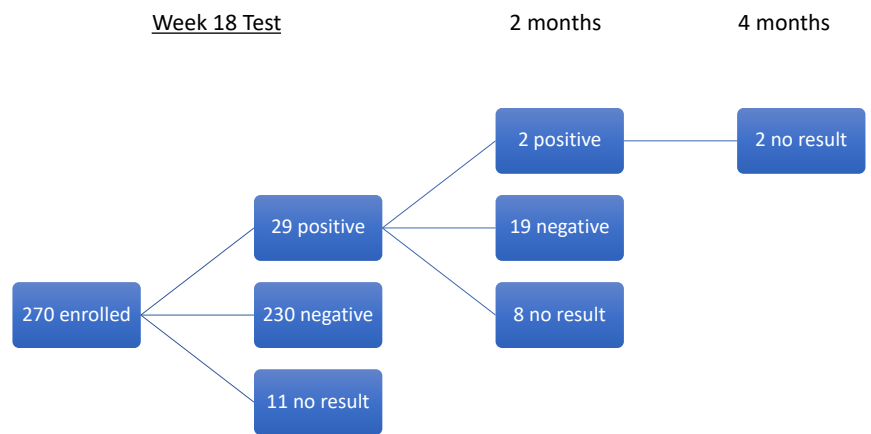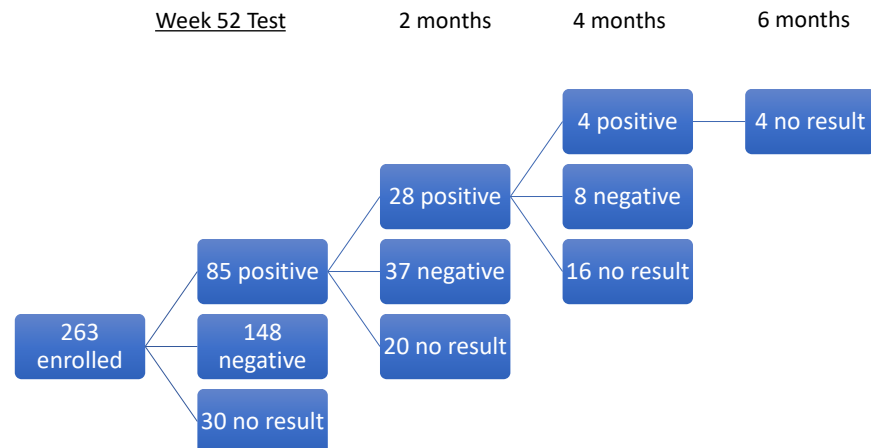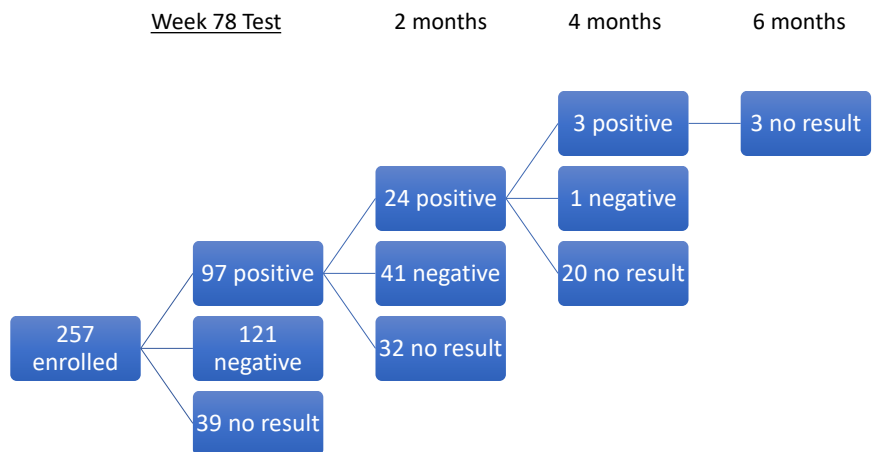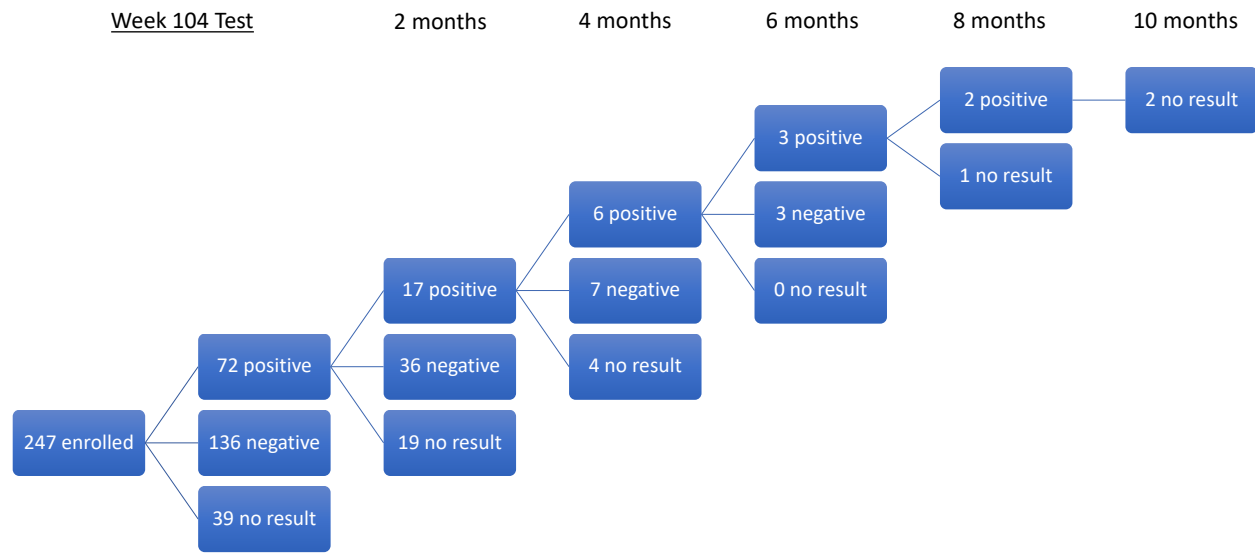

Supplement: SUPPLEMENTARY MATERIAL [file acg-117-167-s002.pdf]
