# Supplementary material for: Small Intestine Bacterial Overgrowth in Bangladeshi Infants Is Associated With Growth Stunting in a Longitudinal Cohort
Source: Am J Gastroenterol. 2021 Oct 25;117(1):167–75. doi: 10.14309/ajg.0000000000001535 (PMC8715995; doi:10.14309/ajg.0000000000001535)
Supplement: SUPPLEMENTARY MATERIAL [file acg-117-167-s003.docx]

**Supplementary Table 2.** The Mean Glucose Hydrogen Breath Test Area Under the H_2_ Curve of Children With (Included in the Pathogen Analysis) and Without (Excluded from the Pathogen Analysis) a Diarrheal Episode in the Three Months Prior to a Scheduled Breath Test.

|  | Mean (SD) GHBT AUC of Children with Diarrhea in the Preceding 3 months (Included in the Pathogen Analysis) | Mean (SD) GHBT AUC of Children without Diarrhea in the Preceding 3 months (Excluded from the Pathogen Analysis) | p value* |
| --- | --- | --- | --- |
| Week 18 | 5.95 (6.43) | 9.98 (14.06) | 0.05 |
| Week 52 | 14.49 (16.23) | 17.99 (15.56) | 0.19 |
| Week 78 | 22.54 (26.10) | 19.26 (15.48) | 0.29 |
| Week 104 | 15.88 (8.95) | 14.97 (12.83) | 0.71 |
| *As determined by Student’s T-Test  Abbreviations: Standard deviation (SD), Glucose hydrogen breath test (GHBT), Area under the H_2_ curve (AUC) | | | |
