# Supplementary material for: Small Intestine Bacterial Overgrowth in Bangladeshi Infants Is Associated With Growth Stunting in a Longitudinal Cohort
Source: Am J Gastroenterol. 2021 Oct 25;117(1):167–75. doi: 10.14309/ajg.0000000000001535 (PMC8715995; doi:10.14309/ajg.0000000000001535)
Supplement: SUPPLEMENTARY MATERIAL [file acg-117-167-s004.docx]

**Supplementary Table 3.** Incidence of Pathogens Detected in Tested Stool Samples (i.e. Children with Diarrhea in the 3 Months Preceding a Glucose Hydrogen Breath Test) at Each Time Point.

|  | Incidence (%) | | | |  |
| --- | --- | --- | --- | --- | --- |
|  | 18 weeks | 52 weeks | 78 weeks | 104 weeks | |
| adenovirus 40/41 | 17.91 | 32.73 | 32.69 | 10.53 | |
| *Aeromonas* | 10.45 | 12.73 | 15.38 | 7.89 | |
| *Ancyclostoma* | 0.00 | 0.00 | 0.00 | 2.63 | |
| *Ascaris* | 0.00 | 0.00 | 3.85 | 10.53 | |
| astrovirus | 5.97 | 38.18 | 19.23 | 21.05 | |
| *Blastocystis* | 0.00 | 7.27 | 11.54 | 31.58 | |
| *Campylobacter* | 28.36 | 61.82 | 73.08 | 73.68 | |
| *C. difficile* | 0.00 | 5.45 | 5.77 | 0.00 | |
| *Cryptosporidium* | 0.00 | 9.09 | 19.23 | 10.53 | |
| *Cyclospora* | 0.00 | 0.00 | 0.00 | 0.00 | |
| EAEC | 59.70 | 94.55 | 73.08 | 81.58 | |
| *E. bieneusi* | 0.00 | 12.73 | 13.46 | 15.79 | |
| *E. histolytica* | 1.49 | 0.00 | 0.00 | 0.00 | |
| *E. intestinalis* | 1.49 | 1.82 | 0.00 | 0.00 | |
| EPEC | 35.82 | 63.64 | 63.46 | 63.16 | |
| ETEC | 29.85 | 60.00 | 65.38 | 60.53 | |
| *Giardia* | 0.00 | 5.45 | 19.23 | 28.95 | |
| *Isospora* | 1.49 | 1.82 | 1.92 | 0.00 | |
| *Necator* | 0.00 | 0.00 | 0.00 | 0.00 | |
| norovirus GI | 4.48 | 7.27 | 9.62 | 5.26 | |
| norovirus GII | 13.43 | 32.73 | 34.62 | 26.32 | |
| *Plesiomonas* | 1.49 | 7.27 | 3.85 | 2.63 | |
| rotavirus | 19.40 | 23.64 | 9.62 | 10.53 | |
| *Salmonella* | 1.49 | 1.82 | 1.92 | 0.00 | |
| sapovirus | 10.45 | 43.64 | 40.38 | 15.79 | |
| *Schistosoma* | 0.00 | 1.82 | 0.00 | 0.00 | |
| *Shigella* | 1.49 | 49.09 | 48.08 | 50.00 | |
| STEC | 0.00 | 1.82 | 3.85 | 2.63 | |
| *Strongyloides* | 0.00 | 0.00 | 0.00 | 0.00 | |
| *Trichuris* | 0.00 | 1.82 | 9.62 | 10.53 | |
| *V. cholerae* | 2.99 | 7.27 | 9.62 | 2.63 | |
| Abbreviations: *Enteroaggregative E. coli* (EAEC), *Enteropathogenic E. coli* (EPEC), *Enterotoxigenic E. coli* (ETEC), *Shiga toxin producing E. coli* (STEC) | | | | | |
