# Supplementary material for: Small Intestine Bacterial Overgrowth in Bangladeshi Infants Is Associated With Growth Stunting in a Longitudinal Cohort
Source: Am J Gastroenterol. 2021 Oct 25;117(1):167–75. doi: 10.14309/ajg.0000000000001535 (PMC8715995; doi:10.14309/ajg.0000000000001535)
Supplement: SUPPLEMENTARY MATERIAL [file acg-117-167-s005.docx]

| **Supplementary Table 1.** False Discovery Rate (FDR) Correction for Multiple Comparisons Applied to p Values Comparing the Glucose-Hydrogen-Breath-Test Area Under the Curve Between Pathogen Exposed and Unexposed Children (Figure 3). | | | |
| --- | --- | --- | --- |
| **Pathogen** | **p value** | **FDR** | **Adaptive FDR** |
| 18 weeks | | | |
| sapovirus | 0.001 | 0.01 | 0.009 |
| EPEC | 0.02 | 0.07 | 0.06 |
| EAEC | 0.02 | 0.07 | 0.06 |
| rotavirus | 0.20 | 0.50 | 0.45 |
| adenovirus 40/41 | 0.25 | 0.50 | 0.45 |
| norovirus GII | 0.40 | 0.67 | 0.60 |
| *Campylobacter* | 0.69 | 0.97 | 0.88 |
| ETEC | 0.81 | 0.97 | 0.88 |
| astrovirus | 0.91 | 0.97 | 0.91 |
| *Aeromonas* | 0.97 | 0.97 | 0.97 |
| 52 weeks | | | |
| EAEC | 0.0001 | 0.002 | 0.002 |
| *C. difficile* | 0.02 | 0.22 | 0.22 |
| EPEC | 0.25 | 0.84 | 0.84 |
| norovirus GII | 0.27 | 0.84 | 0.84 |
| *Plesiomonas* | 0.33 | 0.84 | 0.84 |
| *E. bieneusi* | 0.35 | 0.84 | 0.84 |
| *Blastocystis* | 0.37 | 0.84 | 0.84 |
| *Campylobacter* | 0.37 | 0.84 | 0.84 |
| *Giardia* | 0.43 | 0.86 | 0.86 |
| *Aeromonas* | 0.55 | 0.94 | 0.94 |
| *Cryptosporidium* | 0.59 | 0.94 | 0.94 |
| astrovirus | 0.67 | 0.94 | 0.94 |
| adenovirus 40/41 | 0.68 | 0.94 | 0.94 |
| *V. cholerae* | 0.78 | 0.96 | 0.96 |
| *Shigella* | 0.83 | 0.96 | 0.96 |
| ETEC | 0.87 | 0.96 | 0.96 |
| rotavirus | 0.92 | 0.96 | 0.96 |
| sapovirus | 0.96 | 0.96 | 0.96 |
| 78 weeks | | | |
| *Cryptosporidium* | 0.03 | 0.33 | 0.33 |
| rotavirus | 0.03 | 0.33 | 0.33 |
| adenovirus 40/41 | 0.12 | 0.70 | 0.70 |
| *V. cholerae* | 0.15 | 0.70 | 0.70 |
| *Trichuris* | 0.19 | 0.70 | 0.70 |
| norovirus GI | 0.26 | 0.76 | 0.76 |
| sapovirus | 0.29 | 0.76 | 0.76 |
| *Shigella* | 0.32 | 0.76 | 0.76 |
| ETEC | 0.37 | 0.79 | 0.79 |
| EAEC | 0.46 | 0.86 | 0.86 |
| *Campylobacter* | 0.50 | 0.86 | 0.86 |
| EPEC | 0.60 | 0.86 | 0.86 |
| *Giardia* | 0.60 | 0.86 | 0.86 |
| *Blastocystis* | 0.64 | 0.86 | 0.86 |
| *C. difficile* | 0.72 | 0.88 | 0.88 |
| norovirus GII | 0.78 | 0.88 | 0.88 |
| astrovirus | 0.83 | 0.88 | 0.88 |
| *E. bieneusi* | 0.84 | 0.88 | 0.88 |
| *Aeromonas* | 0.94 | 0.94 | 0.94 |
| 104 weeks | | | |
| *Giardia* | 0.04 | 0.47 | 0.47 |
| *E. bieneusi* | 0.05 | 0.47 | 0.47 |
| ETEC | 0.19 | 0.72 | 0.72 |
| adenovirus 40/41 | 0.24 | 0.72 | 0.72 |
| *Aeromonas* | 0.24 | 0.72 | 0.72 |
| *Cryptosporidium* | 0.24 | 0.72 | 0.72 |
| norovirus GI | 0.35 | 0.83 | 0.83 |
| norovirus GII | 0.37 | 0.83 | 0.83 |
| rotavirus | 0.44 | 0.87 | 0.87 |
| EPEC | 0.54 | 0.90 | 0.90 |
| sapovirus | 0.55 | 0.90 | 0.90 |
| EAEC | 0.69 | 0.90 | 0.90 |
| *Campylobacter* | 0.72 | 0.90 | 0.90 |
| astrovirus | 0.74 | 0.90 | 0.90 |
| *Trichuris* | 0.80 | 0.90 | 0.90 |
| *Ascaris* | 0.80 | 0.90 | 0.90 |
| *Blastocystis* | 0.97 | 0.99 | 0.99 |
| *Shigella* | 0.99 | 0.99 | 0.99 |
| Abbreviations: *Enteropathogenic E. coli* (EPEC), *Enteroaggregative E. coli* (EAEC), *Enterotoxigenic E. coli* (ETEC) | | | |
